# Mechanisms of tumor cell evasion from NK cell-mediated killing and advances in NK cell-based cancer immunotherapy

**DOI:** 10.1016/j.pscia.2026.100109

**Published:** 2026-02-09

**Authors:** Rulin Zheng, Zhuqing Wang, Baodi Zhang, Yiwei Li, Shan Gao, Yuhan Guo, Deping Meng, Xiaodong Mu, Liwei Shao

**Affiliations:** a School of Pharmaceutical Sciences & Institute of Materia Medical, Shandong First Medical University & Shandong Academy of Medical Sciences, Jinan City, 250117, China; b School of Clinical and Basic Medical Sciences, Shandong First Medical University& Shandong Academy of Medical Sciences, Jinan City, 250117, China

**Keywords:** Natural killer cells, Tumor immunology, Tumor microenvironment, Immune evasion, Cancer immunotherapy, CAR-NK therapy

## Abstract

Natural killer (NK) cells represent a critical component of the innate immune system, capable of exerting potent cytotoxic activity against infected and transformed cells. However, tumor cells evolve diverse strategies to evade NK cell-mediated surveillance, including modulation of receptor-ligand interactions, secretion of immunosuppressive cytokines, metabolic disruption, and induction of NK cell exhaustion. In this review, we summarise the mechanisms by which tumors escape NK cell control and highlight recent advances in NK cell-based immunotherapies, including adoptive NK transfer, immune checkpoint blockade, and CAR-engineered NK cells. Ongoing efforts to improve NK cell persistence, infiltration, and functional resilience hold promise for optimising clinical efficacy.

**Table:** Abbreviations

NK	Natural killer
MHC	Major histocompatibility complex
NCRs	Natural cytotoxicity receptors
aKIRs	Activating killer cell immunoglobulin-like receptors
iKIRs	Inhibitory killer cell immunoglobulin-like receptors
TME	Tumor microenvironment
EMT	Epithelial–mesenchymal transition
ECM	Extracellular matrix
TGF-β	Transforming growth factor-β
ILC1	Type 1 innate lymphoid cells
intILC1	Intermediate type 1 innate lymphoid cells
FBP1	Fructose-1,6-bisphosphatase
Id2	Inhibitor of differentiation 2
IRF2	Interferon regulatory factor 2
mTOR	Mammalian target of rapamycin
OXPHOS	Oxidative phosphorylation
GARP	Glycoprotein A repetitions predominant
CAFs	Cancer-associated fibroblasts
EVs	Extracellular vesicles
IDO	Indoleamine 2,3-dioxygenase
PGE2	Prostaglandin E2
FMRP	Fragile X mental retardation protein
ASOs	Antisense oligonucleotides
PTIR1	Putative tumor immunosuppressive receptor 1
PD-1	Programmed cell death protein 1
TIM-3	T cell immunoglobulin and mucin domain 3
TIGIT	T cell immunoreceptor with Ig and ITIM domains
LAG-3	Lymphocyte-activation gene 3
HLA-E	Human leukocyte antigen-E
HLA-G	Human leukocyte antigen-G
Tregs	Regulatory T cells
TAMs	Tumor-associated macrophages
DCs	Dendritic cells
cDC1s	Conventional type 1 dendritic cells
pDCs	Plasmacytoid DCs
ILT7	Immunoglobulin-like transcript 7
AFP	Alpha-fetoprotein
ROS	Reactive oxygen species
AML	Acute myeloid leukemia
NSCLC	Non-small cell lung cancer
HCC	Hepatocellular carcinoma
CRC	Colorectal cancer
OC	Ovarian cancer
GBM	Glioblastoma
HNSCC	Head and neck squamous cell carcinoma
CLL	Chronic lymphocytic leukemia
MDS	Myelodysplastic syndromes
ENKTL	Extranodal NK/T-cell lymphoma
PKLR	Pyruvate kinase liver and red blood cell
PRMT1	Protein arginine methyltransferase 1
HKDC1	Hexokinase domain-containing protein 1
FFA	Free fatty acid
OASL	Oligoadenylate synthetase-like
OXPHOS	Oxidative phosphorylation
PS	Phosphatidylserine
IgSF	immunoglobulin superfamily
PVR	Poliovirus receptor
MICA/B	MHC class I-related chain A/B
sMICA/B	Soluble MICA/B
DISC	Death-inducing signaling complex
CARs	Chimeric antigen receptors
CRS	Cytokine release syndrome
HAIC	Hepatic arterial infusion chemotherapy
ORR	Objective response rate
DCR	Disease control rate
mOS	Median overall survival
PFS	Progression-free survival
MARCO	Macrophage receptor with collagenous structure
cNKs	Conventional NK cells
LrNKs	Liver-resident NK cells
T-MSCs	Tumor-derived mesenchymal stem cells
PDAC	Pancreatic ductal adenocarcinoma
TNF	Tumor necrosis factor
CTCs	Circulating tumor cells
iPSCs	Induced pluripotent stem cells
mAbs	Monoclonal antibodies
CARs	Chimeric antigen receptors
ELISA	Enzyme-linked immunosorbent assay
GVHD	Graft-versus-host disease

## 1 Introduction

NK cells are key effectors of the innate immune system and constitute the first line of defense against invading pathogens and neoplastic cells [1,2]. NK cells can rapidly detect and eliminate infected or transformed cells without prior sensitization and without restriction by the major histocompatibility complex (MHC). In addition to their direct cytotoxicity, NK cells exert indirect antitumor effects by modulating the immune response and enhancing the activity of other effector cells [3]. Accumulating evidence confirms that NK cells suppress a broad spectrum of malignancies, whereas impaired NK-cell activation is tightly associated with poor clinical prognosis. Collectively, these findings establish NK cells as indispensable mediators of antitumor immunity.

The ability of NK cells to recognize and lyse cancer cells is tightly regulated by the balance of signals derived from activating and inhibitory receptors. Prominent activating receptors include NKG2D, natural cytotoxicity receptors (NCRs, including NKp46, NKp30, NKp44 and NKp80), activating killer cell immunoglobulin-like receptors (aKIRs, including KIR2DS1, KIR2DS2, KIR2DS4 and KIR2DL4), CD226, and CD16 [4]. These receptors have been established as critical mediators of anti-tumor responses, and all of which trigger cytotoxic responses upon ligand engagement [[5], [6], [7], [8]]. The inhibitory receptors include inhibitory killer cell immunoglobulin-like receptors (iKIRs, including KIR2DL1, KIR2DL2, KIR2DL3, KIR3DL1 and KIR3DL2) and the heterodimer NKG2A/CD94 receptor [4]. Under physiological conditions, these receptors “educate” NK cells during development and maturation, transmitting dominant inhibitory signals upon ligand recognition. Due to inhibitory receptor–ligand interactions typically exhibit higher binding affinity, inhibitory signals often prevail over activating cues.

In 1986, Kärre proposed the “missing-self” hypothesis, which posits that NK cells preferentially target cells that lack “self” markers-namely, classical MHC class I molecules. Most normal somatic cells constitutively express MHC-I, and recognition by inhibitory receptors prevents NK cells from attacking healthy tissues [4,9]. During oncogenic transformation, however, surface molecule expression is profoundly altered: MHC-I expression may be downregulated, lost, or mutated, whereas ligands for activating receptors such as NCR1 and NKG2D are frequently upregulated. Under these conditions, activating signals dominate, triggering NK cell-mediated cytotoxicity and secretion of proinflammatory cytokines. Nevertheless, as tumors progress from early transformation to clinically evident neoplasia, cancer cells evolve diverse mechanisms of immune evasion. These include aberrant overexpression of oncogenes, downregulation of activating ligands, and establishment of an immunosuppressive tumor microenvironment (TME), collectively leading to NK cell dysfunction and impaired effector capacity [10,11].

To counteract these escape mechanisms, a range of therapeutic strategies has been developed to restore or enhance NK cell functionality. Current NK cell–based immunotherapeutic approaches include: (1) adoptive transfer of ex vivo activated or expanded NK cells to increase both cell numbers and activity in patients; (2) immune checkpoint blockade to reverse NK cell exhaustion; (3) genetic engineering of NK cells with chimeric antigen receptors (CARs) to enhance tumor specificity; and (4) bi- or tri-specific antibody constructs that redirect and potentiate NK cell cytotoxicity. While these approaches have demonstrated encouraging clinical activity, significant challenges remain, including limited persistence, restricted tumor infiltration, and suppression by the TME. Therefore, this review summarizes recent advances in understanding tumor immune evasion from NK cell surveillance and highlights the latest developments in NK cell-based immunotherapies.

## 2 The immunosuppressive tumor microenvironment compromises NK cell activation

Tumor cells can shape an immunosuppressive TME during their interaction with NK cells. This microenvironment induces immune tolerance or drives NK cells into a state of functional exhaustion, thereby diminishing their cytotoxic capacity and impairing effector functions. As a result, NK cells exhibit reduced tumor-killing ability and altered migration dynamics within the TME.

### 2.1 The TME generates immunosuppressive cytokines

**Fig. 1:**
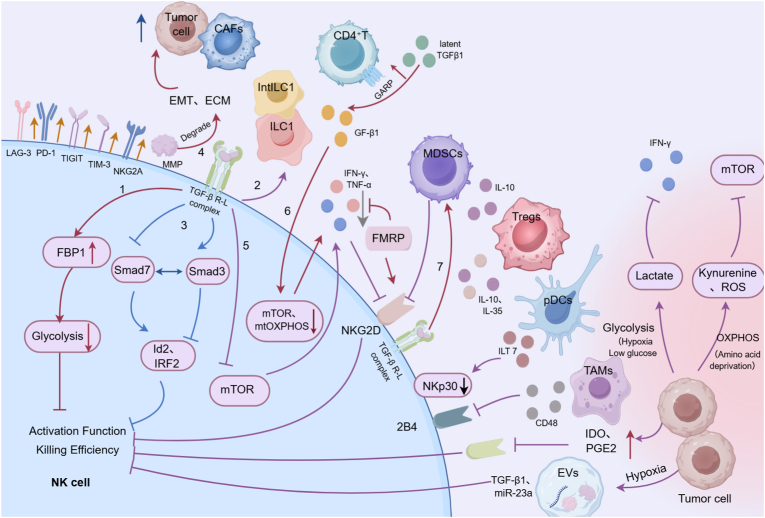
**Factors influencing the exhausted state of NK cells.** The exhaustion of NK cells in the TME is regulated by multiple factors which results in NK cell function impairment due to: the downregulated expression of NK cell-activating receptors (e.g., NKG2D, DNAM-1, NKp30), the reduced secretion of effector cytokines (such as IFN-γ), and the upregulated expression of immunosuppressive molecules (e.g., PD-1, NKG2A, TIGIT, CD96, TIM-3, LAG-3). Additionally, transforming growth factor-β (TGF-β) secreted by tumor cells, cancer-associated fibroblasts (CAFs), and extracellular vesicles (EVs); hypoxic microenvironment; indoleamine 2,3-dioxygenase (IDO); prostaglandin E2 (PGE2); and immunosuppressive cells including macrophages, regulatory T cells (Treg), and myeloid-derived suppressor cells (MDSC) are involved in regulating NK cell exhaustion through multiple pathways.

During the early stages of tumor development, NK cells exhibit potent antitumor activity. However, as the tumor progresses, tumor cell-derived TGF-β accumulates substantially within the TME. TGF-β suppresses NK cell cytotoxicity through multiple mechanisms [12] (Fig. 1):

#### 2.1.1 Metabolic suppression

TGF-β upregulates fructose-1,6-bisphosphatase (FBP1), a key enzyme in gluconeogenesis, in NK cells. Elevated FBP1 expression inhibits glycolysis, thereby reducing NK cell cytotoxic function [13].

#### 2.1.2 Phenotypic conversion

TGF-β drives NK cells toward intermediate type 1 innate lymphoid cells (intILC1) and type 1 innate lymphoid cells (ILC1), both of which have impaired capacity to control tumor growth and metastasis [14].

#### 2.1.3 Smad signaling modulation

TGF-β promotes tumor growth by activating Smad3 signaling while suppressing Smad7. Smad3, as a transcription factor, inhibits NK cell differentiation and maturation by repressing inhibitor of differentiation 2 (Id2) and interferon regulatory factor 2 (IRF2). In contrast, Smad7 exerts opposite effects. In murine melanoma and lung cancer models, pharmacologic induction of Smad7 or inhibition of Smad3 restored Id2 and IRF2 expression, promoted NK cell maturation, and enhanced cytotoxicity [15].

#### 2.1.4 Matrix remodeling

TGF-β stimulates angiogenesis, epithelial-mesenchymal transition (EMT), and extracellular matrix (ECM) degradation by activating the matrix metalloproteinase (MMP) system [16].

#### 2.1.5 mTOR pathway suppression

TGF-β inhibits mammalian target of rapamycin (mTOR) signaling [17], reducing TNF-α and IFN-γ secretion and impairing NKG2D-dependent cytotoxicity, thereby enabling tumor immune escape [18].

#### 2.1.6 Activation via GARP-expressing T cells

Glycoprotein A repetitions predominant (GARP)-expressing CD4^+^ T cells convert latent TGF-β1 to its active form, leading to decreased bone marrow-derived NK cell frequency, reduced mTOR activity, and impaired mitochondrial oxidative phosphorylation(OXPHOS)-all of which collectively suppress NK antitumor activity [19].

#### 2.1.7 Recruitment of immunosuppressive cells

TGF-β facilitates the recruitment and expansion of myeloid-derived suppressor cells (MDSCs) and regulatory T cells (Tregs), which further suppress IFN-γ production and inhibit NKG2D signaling in NK cells through membrane-bound TGF-β. This indirectly induces NK cell exhaustion and functional impairment [20].

Beyond tumor cell-derived TGF-β, cancer-associated fibroblasts (CAFs) also secrete TGF-β to directly inhibit NK cell function (Fig. 1). While some studies suggest that TGF-β signaling plays a suppressive role in pre-neoplastic hepatocyte proliferation during hepatocellular carcinogenesis (HCC) [21]. Sui et al. demonstrated that TGF-β profoundly impairs NK cytotoxicity in HCC, acting as a critical immunosuppressive factor [22]. Importantly, neutralizing antibodies targeting TGF-β signaling effectively reverse NK suppression. Clinically, melanoma patients exhibit significantly higher serum and PBMC levels of TGF-β1 compared to healthy controls, with elevated levels correlating negatively with NK cell activity [23]. Therefore, targeting the TGF-β pathway may restore NK cell-mediated antitumor immunity and offer a novel strategy for cancer immunotherapy.

Other soluble factors also contribute to NK cell suppression in the TME. IDO and prostaglandin E2 (PGE2) are another two critical immunosuppressive mediators [24,25]. IDO, highly expressed in many tumor cell types [[26], [27], [28]], catalyzes tryptophan degradation into kynurenine metabolites. This metabolic reprogramming (i) suppresses T and NK cell cytotoxicity, (ii) induces T cell apoptosis, and (iii) promotes Treg differentiation [29,30]. Inhibition of IDO restores NK cytotoxicity against cervical cancer cells and promotes NK accumulation in the tumor stroma, enhancing antitumor activity [24]. PGE2 further undermines NK function. Studies suggest that NK cells can recruit conventional type 1 dendritic cells (cDC1s) to the TME through secretion of chemokine (C-C motif) ligand 5 (CCL5) and X-C motif chemokine ligand 1 (XCL1) during anti-tumor responses. However, tumor-derived PGE2 not only directly suppresses NK cell activity but also disrupts this immuno-regulatory mechanism by: (i) downregulating chemokine secretion, (ii) reducing chemokine receptor expression, and (iii) consequently impairing cDC1 recruitment to tumor sites-ultimately compromising anti-tumor immunity [25]. In lung cancer, tumor-derived mesenchymal stem cells (T-MSCs) exploit PGE2 to drive NK cell differentiation toward the CD56dim subset, marked by impaired degranulation, reduced expression of activating receptors, and suppressed IFN-γ production in CD56bright subsets [31]. Moreover, IDO and PGE2 synergistically downregulate NKp30, NKp44, and NKG2D expression on NK cells while inhibiting granzyme A release [32]. In HCC, CAFs further exacerbate NK dysfunction through IDO- and PGE2-dependent mechanisms, reducing expression of NKG2D, DNAM-1, and NKp30, lowering cytotoxic molecule secretion, and suppressing overall tumoricidal activity (Fig. 1). Pharmacologic blockade of either IDO or PGE2 restores NK activation and cytotoxic function [33].

Recent discoveries have identified additional immunosuppressive mediators in the TME. Hanahan and colleagues reported that fragile X mental retardation protein (FMRP) suppresses NK function by binding to mRNAs encoding activating receptors (e.g., NKG2D, NKp46) and inhibiting their translation, thereby reducing receptor expression. FMRP may also impair cytokine secretion, including IFN-γ, collectively compromising NK cytotoxicity [34] (Fig. 1). Since NKG2D and NKp46 are core activating receptors required for NK cell recognition of tumor cells [35], the suppression of their expression by FMRP-encoded by the FMR1 gene-directly impairs NK cell-mediated tumor surveillance and cytotoxicity. This represents a novel immunosuppressive mechanism driven by TME. Clinically, FMRP is markedly overexpressed in immune-excluded malignancies such as glioblastoma and high-grade serous ovarian carcinoma, where its levels inversely correlate with intratumoral NK cell infiltration and poor patient prognosis [34]. Although FMRP is an intracellular protein and thus not directly targetable by antibodies, its expression can be modulated through RNA-based strategies, such as antisense oligonucleotides (ASOs) targeting FMR1 mRNA. Recent studies have demonstrated that ASOs can specifically correct aberrant splicing of FMR1 pre-mRNA and effectively downregulate FMRP expression [36]. Collectively, these findings suggest that targeting FMRP may offer a promising approach to reverse NK cell dysfunction, particularly in immune-excluded solid tumors. In November 2023, Yin, Lv, and colleagues identified a novel molecule, putative tumor immunosuppressive receptor 1 (PTIR1), which acts via dual mechanisms: (i) as a transmembrane protein, PTIR1 binds directly to activating receptors such as DNAM-1, blocking ligand engagement, and (ii) PTIR1 recruits SHP-1/2 phosphatases to suppress downstream SYK and ERK signaling, thereby impairing cytotoxic synapse formation and NK degranulation [37]. DNAM-1 is a critical co-stimulatory receptor for NK cell and CD8^+^ T cell mediated antitumor immunity, yet its function is frequently suppressed in the TME through multiple mechanisms [38,39]. Clinically, downregulation of DNAM-1 on peripheral blood NK cells has been consistently observed in patients with solid tumors such as gastric cancer and is associated with disease progression and poor prognosis [40]. Notably, PTIR1 is the first transmembrane inhibitory receptor reported to directly bind and suppress DNAM-1. Its cell surface localization confers high druggability, and the development of blocking antibodies against PTIR1 holds promise for restoring NK cell function-offering a novel therapeutic strategy for “cold” tumors that respond poorly to PD-1/PD-L1 checkpoint inhibitors.

### 2.2 Elevated expression of immune checkpoint molecules on NK cells

Current studies have demonstrated that immune checkpoint molecules are highly expressed on the surface of T cells within the TME, where they play a pivotal role in tumor-induced immunosuppression and immune evasion. Similarly, tumor cells can induce the upregulation of multiple checkpoint molecules on NK cells, including NKG2A, programmed cell death protein 1 (PD-1), T cell immunoglobulin and mucin domain 3 (TIM-3), T cell immunoreceptor with Ig and ITIM domains (TIGIT), CD96, and lymphocyte-activation gene 3 (LAG-3) (Fig. 1). This elevated checkpoint expression drives NK cells into an exhausted state, thereby reducing their antitumor activity.

#### 2.2.1 NKG2A (CD159)

NKG2A (CD159) a heterodimeric inhibitory receptor of the C-type lectin family, is predominantly expressed on NK and NKT cells. It forms a heterodimer with CD94, and ligand binding triggers inhibitory signaling that suppresses NK cell activation and effector function. Elevated NKG2A expression has been reported in acute myeloid leukemia (AML), non-small cell lung cancer (NSCLC), and HCC, correlating with impaired NK cell functionality and poor prognosis [[41], [42], [43]]. In AML, NKG2A overexpression reduces NK cytokine secretion [42]. In HCC, tumor specimens reveal both elevated NKG2A expression and increased levels of its ligand human leukocyte antigen-E (HLA-E). High circulating IL-10 levels in HCC patients have been implicated in driving this upregulation, as IL-10 blockade suppresses NKG2A expression [44]. Similarly, in ovarian cancer, colorectal cancer, and glioblastoma (GBM), the interaction between HLA-E and NKG2A mediates NK cell dysfunction and promotes tumor immune escape [45,46]. Notably, in a GBM mouse model, NKG2A-deficient NK cells exhibit enhanced cytotoxicity against HLA-E^+^ tumor cells, leading to effective suppression of tumor progression and prolonged survival [46].

#### 2.2.2 PD-1

PD-1 initially identified on CD8^+^ T cells, interacts with its ligands PD-L1 and PD-L2, both of which are frequently overexpressed on tumor cells [47]. Aberrantly high PD-1 expression has also been observed on NK cells. PD-1^+^ NK cells represent a terminally differentiated subset with a CD56^dim^/CD16^bright^/KIR^+^/LIR-1^+^/NKG2A^−^/CD57^+^ phenotype, characterized by diminished cytotoxicity, impaired proliferation, and reduced cytokine production. PD-1 blockade partially restores NK activation [48]. Elevated PD-1 expression has been documented in ovarian cancer(OC) ascites, Kaposi's sarcoma, multiple myeloma, head and neck squamous cell carcinoma (HNSCC), and HCC, where PD-1^hi^ NK cells display severe functional impairment [[49], [50], [51]]. Notably, tumor cells can transfer PD-1 to NK cells via SLAM receptor-dependent trogocytosis, a contact-dependent, actin-dependent but transcription-independent process. Tumor-derived PD-1 suppresses NK cytotoxicity, whereas PD-1 blockade reverses this inhibition and slows tumor progression [52].

#### 2.2.3 TIM-3

TIM-3 another inhibitory receptor expressed on T and NK cells, is upregulated in multiple cancers and chronic infections. Peripheral blood NK cells from melanoma patients display elevated TIM-3 expression, which correlates with impaired activation. TIM-3 expression increases progressively during melanoma progression [53]. In gastric cancer, NK cells exhibit significantly higher TIM-3 levels compared to healthy individuals, with advanced-stage patients showing NK cells co-expressing TIM-3 and PD-1 in a profoundly exhausted state [54]. In HCC, both conventional NK cells (cNKs) and liver-resident NK cells (LrNKs) are reduced in frequency and number while displaying markedly elevated TIM-3 expression [55]. Mechanistically, TIM-3 binding to its ligand phosphatidylserine (PS) triggers phosphorylation of cytoplasmic tyrosine residues, enabling TIM-3 to compete with PI3K's p110 subunit for p85 binding. This inhibits downstream Akt-mTORC1 signaling, driving functional exhaustion. TIM-3 blockade restores NK activity and suppresses HCC progression [56]. Furthermore, DHHC9, the enzyme regulating TIM-3 palmitoylation, is highly expressed in tumor-infiltrating NK cells from HCC patients and correlates with poor prognosis. A TIM-3 palmitoylation-blocking peptide has been shown to enhance NK-mediated antitumor activity, representing a potential therapeutic strategy [57]. However, TIM-3's role remains controversial, as some studies suggest its blockade may paradoxically reduce NK cytotoxicity against pancreatic cancer, underscoring the need for further investigation.

#### 2.2.4 TIGIT and CD96

TIGIT and CD96 both members of the immunoglobulin superfamily (IgSF), are inhibitory receptors expressed on T and NK cells. They compete with the activating receptor DNAM-1 for binding to CD155 (PVR) and CD112 (Nectin-2), with higher affinity than DNAM-1 [58]. CD96 is constitutively expressed on resting NK cells, while TIGIT expression is induced upon activation. These receptors synergistically suppress NK function in the TME: TIGIT attenuates cytotoxicity, whereas CD96 inhibits IFN-γ secretion, collectively counterbalancing DNAM-1 activation [59,60]. In colorectal cancer, TIGIT^hi^ NK cells show severely compromised functionality and associate with advanced disease, while TIGIT blockade reverses exhaustion and delays tumor growth [61]. Reduced TIGIT expression also decreases NK susceptibility to MDSC-mediated suppression [62]. In HCC, CD96^+^ NK cells secrete less IFN-γ and TNF-α, with concomitant reductions in T-bet, perforin, and granzyme B expression. A higher frequency of CD96^+^ NK cells correlates with shorter disease-free survival [63]. Promisingly, dual targeting of TIGIT/CD96 has shown preclinical success: the Fc-enhanced bispecific antibody BMS-986442 synergistically enhances both NK- and T cell-mediated antitumor immunity [64].

#### 2.2.5 LAG-3

LAG-3 another member of IgSF. It is now present on both activated and exhausted NK subsets [65]. Its checkpoint function depends on ligand-induced ubiquitination of K498, which disrupts FSALE motif localization and activates inhibitory signaling [66]. In murine models, LAG-3 deficiency impairs NK-mediated clearance of certain tumors but does not affect lysis of MHC-I-mismatched targets [67]. Clinically, in chronic lymphocytic leukemia (CLL), elevated LAG-3 and soluble LAG-3 (sLAG-3) correlate with adverse cytogenetics and poor prognosis. Blockade with relatlimab, a humanized anti-LAG-3 antibody, enhances NK activity [68]. In HCC patients treated with sorafenib, co-expression of LAG-3 and CD16 on NK cells reduces drug efficacy and associates with early-onset cutaneous toxicity [69]. However, sLAG-3 binding to MHC-II appears to have no impact on NK cytotoxicity [70]. These findings highlight LAG-3 as an emerging target in NK biology, though its functional consequences warrant further study.

### 2.3 Immunosuppressive cells negatively regulate NK cell activity

The TME harbors multiple immunosuppressive cell populations that inhibit NK cell activity through diverse mechanisms.

#### 2.3.1 Tregs

Tregs suppress NK function via several pathways. First, Tregs exert direct inhibition through surface expression of CTLA-4, which reduces production of activating cytokines such as IFN-γ and TNF-α, thereby impairing NK-mediated tumor cytotoxicity [71]. Second, Tregs secrete large amounts of immunoregulatory cytokines (IL-10, IL-35, and TGF-β), creating a strongly immunosuppressive microenvironment that dampens NK activation and effector function [72] (Fig. 1). Third, Tregs regulate NK function via an IFN-γ-STAT1-IFITM3 negative feedback loop. Notably, genetic deletion of IFITM3 enhances NK-mediated antitumor activity and IFN-γ secretion, disrupting the immunosuppressive equilibrium within the TME [73].

#### 2.3.2 MDSCs

MDSCs represent another critical immunosuppressive population that facilitates tumor progression by both suppressing effector immune cell function and promoting angiogenesis and invasion [74]. Activated MDSCs recruit Tregs into the TME through TGF-β and IL-10 secretion, thereby establishing an immunosuppressive niche that simultaneously impairs NK cytotoxicity and promotes tumor proliferation [75] (Fig. 1). In addition, MDSCs suppress NK proliferation through L-arginine depletion mediated by constitutive arginase expression [76]. Their immunosuppressive activity is further enhanced through the CD300ld-STAT3-S100A8/A9 signaling axis. Moreover, breast cancer-derived exosomes induce expression of macrophage receptor with collagenous structure (MARCO), which promotes differentiation of monocytic MDSCs (M-MDSCs). Collectively, these mechanisms profoundly suppress NK activity [77,78].

#### 2.3.3 Tumor-associated macrophages (TAMs)

Tumor-associated macrophages (TAMs) derived from infiltrating macrophages, represent another abundant immune population within tumors, sometimes comprising up to 50% of the tumor mass [[79], [80], [81]]. TAMs promote tumor growth, angiogenesis, metastasis, and invasion [82], while also releasing immunoregulatory mediators that directly suppress NK function [83]. CD68^+^ monocytes/macrophages and NK cells preferentially accumulate in peritumoral stroma rather than intratumoral regions, and high stromal macrophage density correlates with impaired NK function, reduced NK infiltration, and functional exhaustion. In HCC, TAM-mediated NK suppression occurs primarily via CD48-2B4 blockade, reflecting high CD48 expression, rather than through NKG2D/NKp30 pathways [84] (Fig. 1). Furthermore, TREM2^+^ TAMs suppress CXCL9 and IL-15 signaling, markedly impairing NK infiltration and effector function. This mechanism has been validated across multiple cancer types, underscoring a novel pathway by which TAMs regulate NK surveillance [85].

#### 2.3.4 Dendritic cells (DCs)

Dendritic cells (DCs) also play a pivotal role in shaping NK activity. The TME disrupts NK-dDC crosstalk through multiple pathways, fostering immune tolerance and functional suppression [86]. For example, breast cancer and melanoma cells induce immunoglobulin-like transcript 7 (ILT7) expression on plasmacytoid DCs (pDCs), which reduces IFN-α secretion and increases TGF-β production. This downregulates activating receptors (e.g., NKp30 and NKG2D) on NK cells, diminishing NK cytotoxicity toward immature DCs, leading to their accumulation and promoting tumor progression [87] (Fig. 1). Similarly, alpha-fetoprotein (AFP), a liver tumor antigen, impairs NK activation by suppressing DC-derived IL-12 secretion, highlighting its immunomodulatory role in HCC [88]. However, DC impairment can also reinforce NK suppression: PGE2-treated mature DCs display reduced NK recruitment and IFN-γ induction, while Tregs further disrupt NK-DC interactions by downregulating IL-15Rα expression on DCs, ultimately impairing immune synapse formation [72,89].

### 2.4 Immuno-metabolic dysregulation in the TME suppresses NK cell activation and function

Mounting evidence suggests that cancer, like hypertension and diabetes, can be fundamentally considered a metabolic disorder. OXPHOS and glycolysis are two fundamental energy-producing pathways that normally function in a coordinated manner to meet cellular demands. Glycolysis, which occurs in the cytoplasm, rapidly breaks down glucose to generate ATP and metabolic intermediates essential for biosynthesis, but with low energy efficiency. In contrast, OXPHOS, localized in mitochondria, is highly efficient and serves as the primary source of ATP under oxygen-replete conditions, but at a slower rate. In healthy cells, these pathways are dynamically balanced to ensure stable energy and biomass supply across varying physiological states.

However, during tumor initiation and progression, tumor cells and immune cells coexist within the same microenvironment and compete for limited nutrients, including glucose, amino acids, and oxygen [72]. Tumor cells often exhibit the Warburg effect-preferentially engaging in high-rate glycolysis even under aerobic conditions. This metabolic reprogramming leads to lactate accumulation, glucose depletion, and hypoxia in the tumor microenvironment, all of which directly impair NK cell cytotoxicity, cytokine production, and metabolic fitness. Moreover, certain tumor-derived metabolites can act as signaling molecules that further disrupt NK cell metabolism and effector functions [90] (see Table 1).

**Table 1:** Key metabolic alterations in the TME and their suppressive effects on NK cell function.

Metabolic Pathways	Key Metabolite/Condition	Major Effects on NK Cells
Glycolysis (Fig. 1)	Lactate	Inhibits NK cell cytotoxicity and IFN-γ secretion [91]; induces TME acidosis, further impairing immune cell function [92]; promotes lactylation of intracellular proteins in NK cells, disrupting mitochondrial function and compromising antitumor activity [91].
	Low glucose	Tumor cells consume large amounts of glucose, limiting substrate uptake by NK cells via GLUT1 [93]; impairs glycolysis and oxidative phosphorylation, thereby suppressing proliferation, activation, cytokine production, and cytotoxic capacity [94].
	Hypoxia	Activates HIF-1α signaling, impairing NK cell metabolic adaptation; reduces IFN-γ, granzyme B, and perforin release [95,96]; significantly downregulates expression of key activating receptors such as NKG2D, NKp30, and CD16, weakening tumor recognition and elimination ability [97,98].
OXPHOS and Related Pathways OXPHOS (Fig. 1)	Reactive Oxygen Species (ROS)	Induces intracellular oxidative stress, causing mitochondrial structural and functional damage; promotes NK cell apoptosis and dysfunction, with particularly pronounced effects in the CD56bright subset in liver metastases [92].
	Amino acid deprivation	Essential amino acids such as tryptophan are consumed by tumor cells or immunosuppressive cells (e.g., Tregs), inhibiting mTOR signaling and destabilizing mitochondrial homeostasis, ultimately suppressing NK cell activation, proliferation, and effector functions [99].
	Kynurenine	As a metabolite of tryptophan, kynurenine directly acts on NK cells to suppress their proliferation and significantly impair their cytotoxic killing activity [100].

In summary, tumor cells exploit dysregulated OXPHOS and glycolytic metabolism to establish a profoundly immunosuppressive metabolic microenvironment. Within this niche, multiple metabolic products act in concert, severely weakening the anti-tumor immune function of NK cells in various aspects, including energy deprivation, signal interference, and direct toxicity. A deeper understanding of these metabolic checkpoints may inform the development of novel immunotherapeutic strategies, and reversing such metabolic suppression represents a promising avenue for next-generation cancer immunotherapy.

### 2.5 Tumor-derived extracellular vesicles suppress NK cells activation

EVs have emerged as pivotal immunomodulatory mediators in tumorigenesis and cancer progression [101]. By interacting with recipient cell surfaces or transferring bioactive cargo, EVs facilitate intercellular communication and reprogramming. Within the TME, tumor-derived EVs can be internalized by neighboring tumor cells, stromal cells, and immune cells, thereby profoundly influencing antitumor immune responses [102].

More and more evidence indicates that tumor-derived exosomes and microvesicles in the serum of cancer patients contain abundant membrane-associated TGF-β1 secreted by tumor cells [103,104]. The immunosuppressive effects of most tumor-derived EVs on NK cell function are primarily attributed to this cytokine [105]. Serum from patients with AML contains abundant EVs that carry membrane-bound TGF-β1, which suppresses NKG2D expression and impairs the cytotoxic function of NK cells [106]. Similarly, pancreatic cancer-derived EVs express high levels of TGF-β1 and potently inhibit NK cell activation and effector functions, including reduced cytokine production and diminished cytotoxicity. While it was also found that the ability of NK cells to take up glucose is also inhibited [107] (Fig. 1).

Hypoxia further amplifies the immunosuppressive activity of tumor-derived EVs. Berchem et al. demonstrated that EVs isolated from tumor cells cultured under hypoxic conditions suppress NK cytotoxicity through the combined action of TGF-β1 and miR-23a. Importantly, miR-23a acts as an independent immunoregulatory factor by directly inhibiting CD107a expression, a key degranulation marker required for NK-mediated killing [108].

HCC provides another example of EV-mediated suppression. HCC progression is characterized by profound metabolic and immune remodeling of the TME [109]. In a diethylnitrosamine (DEN)-induced HCC mouse model, hepatocyte-specific deficiency of FBP1 resulted in reduced NK infiltration and impaired cytotoxicity. Mechanistically, loss of FBP1 suppressed an EZH2-dependent transcriptional program, thereby downregulating pyruvate kinase liver and red blood cell (PKLR) expression. The subsequent reduction in PKLR cargo within hepatocyte-derived EVs diminished NK activity upon vesicle uptake, ultimately accelerating HCC progression [110].

In colorectal cancer (CRC), EVs have been shown to promote hepatic metastasis by functionally impairing LrNKs. CRC-derived EVs deliver TGF-β1 and miR-21-5p to hepatic NK cells, where they suppress NKG2D and CD226 expression, inhibit mTOR signaling, and impair cytotoxicity and IFN-γ secretion. Concurrently, these vesicles recruit MDSCs, establishing a pro-metastatic niche [111].

Systematic analyses further demonstrate that tumor-derived exosomes suppress NK activity through multiple synergistic mechanisms. Beyond TGF-β1 and miRNA-mediated suppression, exosomal surface immune checkpoint molecules such as PD-L1 and Galectin-9 directly engage inhibitory receptors on NK cells. Additionally, cargo metabolic enzymes including IDO1 and LDHA deplete essential amino acids and disrupt mitochondrial metabolism, further impairing NK activation [112].

In summary, tumor-derived EVs employ cytokines, miRNAs, immune checkpoint ligands, and metabolic enzymes to orchestrate multifaceted suppression of NK cells. These findings underscore the central role of EVs in shaping immune evasion and highlight the need for continued investigation into EV-targeted therapeutic strategies.

## 3 Tumor cells promote immune escape by regulating the expression level of receptor corresponding ligand

The activation status of NK cells is determined by the balance of signals generated through the interplay between activating and inhibitory receptors on their surface. These receptors recognize corresponding ligands on target cells, enabling NK cells to distinguish healthy from aberrant cells and to integrate these signals for effective immune surveillance [113]. However, tumor cells employ diverse strategies to evade NK detection and elimination. Through processes such as “immunoediting” or selective pressure, they reduce their immunogenicity, thereby impairing NK cell recognition and cytotoxicity.

### 3.1 Tumor cells undergo immune editing to reduce immunogenicity

The immune system exerts a dual role in both surveilling and eliminating tumor cells. In 2002, tumor biologist R.D. Schreiber introduced the concept of cancer immunoediting, proposing that the immune system not only eradicates tumor cells but also shapes their evolution. Through this process, tumor cells undergo alterations in immunogenicity that enable malignant progression, resistance to immune responses, and eventual evasion of immune surveillance, thereby promoting tumor growth and dissemination [114].

#### 3.1.1 PRMT1-MYC axis

**Fig. 2:**
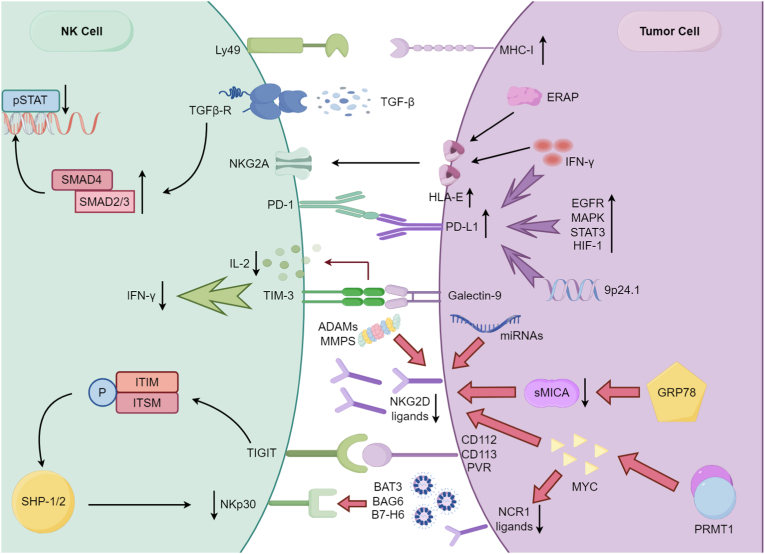
**Tumor cells evade the immune system by reducing the recognition ability of NK cells.** Tumor cells evade the immune surveillance of NK cells by altering the levels of their ligand molecules through three pathways. First, tumor cells actively reduce their own immunogenicity through the immunoediting process, characterized by downregulation of activating receptor ligands (e.g., NKG2D/NCR1 ligands) and upregulation of inhibitory ligands (e.g., MHC-I, PD-L1), thereby reshaping their surface molecular characteristic. Second, tumor cells overexpress various inhibitory receptor ligands on their surface, including PD-L1, HLA-E, and CD155, etc. These molecules bind to inhibitory receptors (e.g., PD-1, NKG2A, TIGIT) on NK cells, and transmit negative regulatory signals to suppress the activation of NK cells. Finally, tumor cells release soluble ligand molecules (e.g., sMICA, sBAG6) through ADAMs/MMPs-mediated proteolytic cleavage or exosomal pathways. These soluble ligands act as “decoys” that competitively bind to activating receptors (e.g., NKG2D, NKp30) on NK cells, blocking recognition and activation signals. These synergistic mechanisms collectively disrupt the balance between activating and inhibitory signals in NK cells, ultimately enabling tumor cells to successfully evade immune surveillance.

Protein arginine methyltransferase 1 (PRMT1) is recognized as an oncogene in multiple cancer types due to its direct interaction with the oncoprotein MYC. PRMT1 catalyzes asymmetric dimethylation of arginine residues (ADMA modification) on MYC, enhancing its stability and suppressing degradation. Zhou et al. showed that PRMT1 modifies MYC at R346, thereby promoting liver cancer progression. Mechanistically, the PRMT1-MYC axis mediates immune editing by suppressing NKG2D/NCR1 ligand expression, upregulating MHC-I, and reducing immune infiltration via inhibition of the cGAS-STING pathway (Fig. 2). Clinically, high PRMT1 expression correlates with decreased CD8^+^ T cell and NK cell infiltration and poor survival, underscoring its central role in immunoediting [115].

#### 3.1.2 Metabolic reprogramming in ovarian cancer

In OC, hexokinase domain-containing protein 1 (HKDC1) drives lipid metabolic reprogramming by stabilizing G6PC/G6PC2, thereby increasing PD-L1 expression and suppressing T cell function. HKDC1 upregulates lipid synthesis enzymes (ACC1, FASN, SCD1), leading to free fatty acid (FFA) and cholesterol accumulation. This metabolic shift impedes T cell proliferation and IFN-γ secretion via the PD-1/PD-L1 axis. Thus, the HKDC1-G6PC/G6PC2 pathway enables immune evasion through dual mechanisms: metabolic suppression of T cell activity and reinforcement of immune checkpoint signaling [116].

#### 3.1.3 OASL-mediated immune evasion in pancreatic cancer

In pancreatic ductal adenocarcinoma (PDAC), oligoadenylate synthetase-like (OASL) protein facilitates MHC-I degradation via the NBR1-mediated autophagy-lysosomal pathway. Xing et al. reported that OASL overexpression correlates negatively with MHC-I levels, while OASL knockout enhances CD8^+^ T cell infiltration and suppresses tumor growth. Mechanistically, OASL (1) forms a complex with NBR1 to promote MHC-I ubiquitination, (2) directs MHC-I to the autophagy pathway via early endosomes, and (3) accelerates LC3-II-dependent fusion of autophagosomes with lysosomes, culminating in MHC-I degradation. This process prevents antigen presentation and blunts CD8^+^ T cell cytotoxicity. Importantly, OASL is dynamically upregulated during PDAC progression, further promoting immune escape [117].

#### 3.1.4 NKG2D/NCR1 ligand regulation

Raju and colleagues observed that B lymphoma cells from NKG2D-deficient mice expressed high levels of NKG2D ligands, whereas expression was minimal in wild-type mice [118]. Similar findings were reported in prostate cancer and Eμ-Myc-driven lymphoma, where tumors from wild-type mice displayed reduced NKG2D ligand expression compared to NKG2D-deficient mice. In sarcomas, subsets of tumor cells expressed either high or low levels of H60a, an NKG2D ligand. Transplantation of H60a^high^ cells into RAG2^−/−^ mice resulted in reduced H60a expression, highlighting selection pressure during tumor development [119].

#### 3.1.5 NCR1/NKp46 immunoediting

Elboim et al. demonstrated that tumors from NKp46-deficient mice expressed NCR1 ligands, whereas wild-type tumors lacked them, suggesting immunoselective downregulation.

#### 3.1.6 Epigenetic silencing and stemness

Huntington et al. further showed that tumor cells downregulate NKG2D/NCR1 ligand expression via promoter hypermethylation. Single-cell sequencing revealed enrichment of stem-like tumor subpopulations driven by Wnt/β-catenin signaling. Notably, MHC-I upregulation was accompanied by increased PD-L1 expression, creating a cooperative immune checkpoint evasion mechanism. Clinically, MHC-I^high^ tumors exhibited 40–60% lower response rates to PD-1 inhibitors, supporting the rationale for combined therapies targeting NK cells [120].

#### 3.1.7 MHC-I upregulation and Ly49 receptors

Under homeostatic conditions, most healthy cells express MHC-I molecules, which engage inhibitory NK receptors and prevent autoimmunity. Consequently, NK cells preferentially eliminate MHC-I-deficient cells. The Ly49 receptor family plays a critical role in NK development and recognition of MHC-I loss (Fig. 2).Tu et al. demonstrated that in melanoma, sarcoma, and B cell lymphoma, tumors from wild-type mice exhibited elevated MHC-I alleles (H-2K^b^, H-2D^b^) following NK immunoediting, compared to Ly49-deficient NKCKD mice. By upregulating MHC-I, tumor cells escape NK recognition by masquerading as “self” cells [121].

Collectively, NK cells exert strong selective pressure on tumor populations, driving immunoediting processes that include downregulation of activating ligands, epigenetic silencing of ligand genes, metabolic reprogramming, and compensatory upregulation of inhibitory signals such as MHC-I and PD-L1. These adaptations reduce tumor immunogenicity and facilitate immune evasion. A deeper understanding of NK cell-driven immunoediting mechanisms is essential for advancing NK-based immunotherapies.

### 3.2 Ligands for inhibitory receptors highly expressed in tumor cells

The evidence summarized above indicates that NK cells within the TME display increased expression of diverse immune checkpoint molecules. In parallel, tumor cells upregulate the ligands for these checkpoints during proliferation, which diminishes NK cell activation and functionality. Tumor cells can establish immunosuppressive mechanisms by engaging inhibitory ligands on their surfaces with NK cell receptors, thereby inducing immune tolerance and compromising NK cell function [122].

The known ligands for PD-1 are PD-L1 and PD-L2, which are highly expressed on tumor cells and associated with unfavorable prognoses [[123], [124], [125]]. These ligands engage PD-1 to impair antigen presentation signaling and disrupt immune cascade responses. Tumor cells upregulate PD-L1 expression through several mechanisms:

(1) **Signal transduction pathways:** Activation of EGFR, MAPK, or PI3K-Akt pathways, overexpression of STAT3, and induction of HIF-1 transcription factors. For example, IL-10 binding to IL-10R1/IL-10R2 activates JAK1/TYK2 kinases, resulting in STAT3 phosphorylation. Phosphorylated STAT3 dimerizes, translocates to the nucleus, binds promoter regions, and induces anti-apoptotic and immunosuppressive functions [126]. In colorectal cancer liver metastasis models, IL-10 secreted by Foxp3^+^ Tregs acts on myeloid cells via IL-10Rα, strongly activating JAK1/STAT3 signaling, which in turn enhances PD-L1 expression [127].
(2) **Gene amplification:** Chromosomal region 9p24.1 contains the PD-L1 (CD274) and PD-L2 (PDCD1LG2) genes, as well as JAK2. Amplification of this locus increases PD-L1 expression and promotes immune evasion via the PD-1/PD-L1 axis. JAK2 also regulates IFN-γ signaling, thereby modulating PD-L1 levels and shaping the TME [128].
(3) **Viral induction:** EBV-positive gastric and nasopharyngeal carcinomas exhibit high PD-L1 expression even without 9p24.1 amplification.
(4) **Epigenetic and inflammatory regulation:** PD-L1/PD-L2 expression can be induced by inflammatory cytokines, particularly IFN-γ. In our studies of liver cancer, PD-L1 expression on tumor cells correlated with impaired NK cell function [43,129].

Notably, PD-L1 is expressed not only on tumor cells but also on NK cells within the TME [130]. Recent findings suggest that PD-L1^+^ NK cells exhibit heightened activation, underscoring the need for deeper investigation into the dual role of PD-L1 within the TME. Accordingly, therapeutic use of PD-L1 inhibitors should be tailored to the immunological context (Fig. 2).

TIM-3 ligands-including Galectin-9, PS, HMGB1, and CEACAM-1-play crucial roles in regulating NK activity. In AML, Galectin-9 is markedly overexpressed in tumor cells, and its binding to TIM-3 strongly suppresses NK cell activity and IL-2 secretion [131]. Elevated TIM-3 expression has also been reported in HPV-positive cervical cancer, where it correlates with poor differentiation, lymphatic metastasis, and reduced five-year survival [132]. Interestingly, blocking Galectin-9 in AML reduced IFN-γ secretion by NK cells during co-culture, highlighting the complex interplay between TIM-3 and its ligands. A more comprehensive understanding of TIM-3-ligand interactions is essential for elucidating their precise roles in NK cell regulation (Fig. 2).

The natural ligand for NKG2A in humans is HLA-E, while in mice it is Qa-1b (the HLA-E homolog). Human leukocyte antigen G (HLA-G) has also been proposed as an NKG2A ligand [133]. Under physiological conditions, HLA-E is expressed at low levels; however, it is frequently upregulated in tumor cells, where it suppresses NK cytotoxicity and facilitates immune evasion [[134], [135], [136], [137], [138]]. The analysis of 261 patients with diffuse glioma revealed that the expression level of HLA-E in high-grade glioma was significantly elevated compared to that in low-grade glioma. Increased levels of HLA-E mRNA were correlated with poorer progression-free survival and overall survival in patients with low-grade glioma [139]. Consequently, elevated HLA-E expression correlates with poor clinical outcomes [133]. IFN-γ has been shown to induce HLA-E upregulation [[140], [141], [142]], potentially via interactions with tumor cell epithelial adhesion molecules [143]. HLA-E activity is also regulated by ERAP, a peptide-loading enzyme, which modulates NKG2A signaling through SHP-1/2 phosphatase recruitment (Fig. 2). Inhibition of ERAP disrupts the HLA-E-NKG2A axis, restoring NK-mediated killing of circulating tumor cells [144]. In addition, HLA-G is upregulated in several tumor types, further contributing to NK cell suppression [145].

TIGIT ligands include CD112, CD113, and CD155 (also known as PVR, Necl-5, or Tage4) [146] (Fig. 2). CD155, an IgSF adhesion molecule, contributes to immune evasion by driving CD8^+^ T cell exhaustion via the PI3K/AKT/NF-κB pathway, reducing IFN-γ and granzyme B secretion and correlating with diminished T cell infiltration [147]. TIGIT competes with the activating receptor CD226 for binding to CD155, with substantially higher affinity, thereby blocking CD226-mediated NK activation and transmitting inhibitory signals. This results in reduced NK cytotoxicity, degranulation (e.g., CD107a expression), and cytokine production [148].

High CD155 expression correlates with colorectal cancer progression and serves as a potential prognostic biomarker. Genetic deletion of CD155 in colon cancer cells inhibits migration and invasion, downregulates FAK, Src, and MMP-2 expression, induces G1 cell-cycle arrest, and enhances apoptosis [149]. Elevated CD155 not only drives proliferation and metastasis but also contributes to immune evasion. In NSCLC and breast cancer, co-expression of PD-L1 and CD155 correlates with survival outcomes, highlighting their combined clinical significance for prognosis.

### 3.3 Tumor cells release ligands for soluble activating receptors

In addition to immunoediting, tumor cells employ alternative strategies to impair NK cell activity by reducing the surface expression of activating ligands. One key mechanism involves the release of soluble ligands that act as molecular decoys for activating receptors on NK cells, thereby blocking receptor–ligand interactions and attenuating immune surveillance [150,151]. This process is largely mediated by metalloproteinases, including ADAM10, ADAM17, and MMP14 [152]. Elevated levels of soluble activating ligands in patient serum have been associated with advanced disease and poor clinical outcomes [[153], [154], [155]].

NKG2D ligands are particularly susceptible to this evasion strategy (Fig. 2).

(1) **Proteolytic shedding:** Metalloproteinases such as ADAM10 and ADAM17 cleave membrane-bound MICA/B (mMICA/B) from tumor cell surfaces, generating soluble MICA/B (sMICA/B). These soluble forms bind NKG2D receptors on NK cells, preventing their engagement with tumor-associated ligands and impairing NK cytotoxicity [156,157].
(2) **Post-translational modifications:** Palmitoylation of MICA influences its localization to membrane microdomains and facilitates shedding. In HCC, the molecular chaperone GRP78 reduces MICA/B expression via post-transcriptional regulation, further limiting NK cell recognition [158]. Additionally, cleavage of integrin metalloproteinase 9 leads to partial loss of the MICA ectodomain and sMICA generation. Elevated serum sMICA levels correlate with portal vein tumor thrombus in HCC, promote NKG2D internalization and degradation, and impair NK cell antitumor activity [159,160].
(3) **MicroRNA regulation:** Tumor-derived miRNAs downregulate NKG2D ligands (Fig. 2). For example, miR-183 (induced by TGF-β), miR-20a, miR-146b-5p, and miR-25/93/106b suppress MICA expression [[161], [162], [163], [164]].
(4) **Exosome-mediated release:** Exosomes from prostate, ovarian, and melanoma cells carry NKG2D ligands, serving as decoys that inhibit NK function [[165], [166], [167], [168]]. Importantly, a dynamic balance exists between proteolytic shedding and exosomal release of NKG2D ligands such as BAG6 [169]. Interestingly, in mice, soluble ULBP1 (MULT1) has been reported to enhance NK activity by mitigating chronic NKG2D stimulation, suggesting that not all soluble ligands are suppressive [170].

Other activating receptor ligands are also targeted. **NKp30 ligands:** BAT3/BAG6 and B7-H6 are frequently released in soluble or exosomal forms (Fig. 2). Soluble BAG6 impairs NK cytotoxicity, whereas exosomal BAG6 enhances NK activity through NKp30 engagement [171,172]. Soluble B7-H6 has been detected in septic patient serum and associated with higher mortality rates [173,174]. Tumor-derived ADAM10 and ADAM17 cleave B7-H6 from the cell surface, thereby weakening NKp30-mediated antitumor responses [175].

In summary, tumor-derived soluble ligands for activating receptors-particularly NKG2D and NKp30-serve as decoys that suppress NK function and promote immune evasion. These findings underscore the therapeutic potential of targeting ligand shedding and exosomal release to restore NK cell–mediated antitumor activity.

## 4 Enhanced anti-apoptotic capacity of tumor cells and their ability to induce NK-cell apoptosis

### 4.1 Tumor cells resist apoptosis mediated by NK cells granules

NK cells eliminate malignant cells primarily through the exocytosis of perforin and granzymes, which activate caspase cascades in target cells, induce DNA fragmentation, and trigger apoptosis. However, during tumor progression, cancer cells frequently acquire resistance to granule-mediated apoptosis by disrupting apoptotic pathways or neutralizing effector molecules delivered by NK cells [176].

**Fig. 3:**
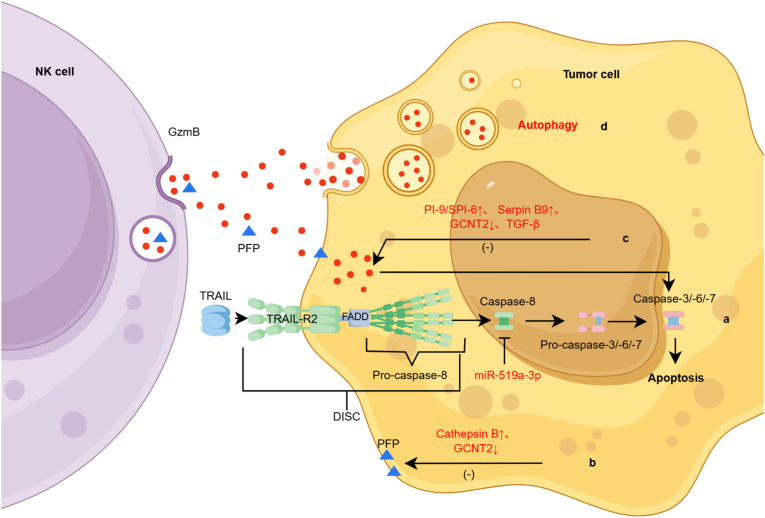
**Tumor cells resist apoptosis mediated by Granzymes released from NK cells.** Multilayered tumor-intrinsic resistance to NK cell-mediated Granzyme/Perforin cytotoxicity. (**a:** miR-519a-3p suppresses Caspase-8, Caspase-7, and TRAIL-R2 expression, conferring resistance to both Granzyme-B- and TRAIL-induced apoptosis; **b:** Tumor cells high expression of Cathepsin B leads to perforin inactivation, which can also be reduced by downregulating GCNT2 to reduce granulocytin B and perforin mediated apoptosis; **c**: Tumor cells can reduce the release of granulase B, high expression of Serpin B9, and upregulation of Pl-9/SPl-6 by TGF-β signaling pathway to inhibit granulase B-mediated apoptosis; **d:** Autophagy-induced granzyme inactivation.).

Genetic alterations in apoptotic mediators are one major resistance mechanism. In advanced gastric cancer and HCC, somatic loss-of-function mutations in Caspase-8 occur in 10% and 13% of patients, respectively [177,178]. Mutations in Caspase-3 and Caspase-9 have also been reported across multiple tumor types [179,180]. In breast cancer, miR-519a-3p suppresses Caspase-8, Caspase-7, and TRAIL-R2 expression, conferring resistance to both Granzyme-B- and TRAIL-induced apoptosis (Fig. 3a) [181]. Tumors also upregulate anti-apoptotic proteins such as Bcl-2, members of the IAP family, and apoptosis regulators including Bax and PCAF, although the extent to which these changes alter NK-mediated cytotoxicity remains incompletely defined [[182], [183], [184], [185]].

Tumor cells can also act directly on NK effector molecules. For example, AML blasts prevent perforin binding to their surface, thereby reducing NK-mediated killing [186]. Many tumors overexpress Cathepsin B, a protease that degrades extracellular perforin and blocks pore formation (Fig. 3b) [187,188]. Similarly, several cancers express granzyme inhibitors:

(1) **Serpin B9** in lymphoma cells blocks Granzyme-B–mediated apoptosis (Fig. 3c) [189].
(2) **Serpin B4** in squamous cell carcinoma inhibits Granzyme M [190].
(3) Bladder cancer cells downregulate **GCNT2**, conferring resistance to perforin- and Granzyme-B–mediated killing (Fig. 3b and c) [191].

Hypoxia further enhances resistance through autophagy-mediated degradation of Granzyme B, reducing NK-mediated cytotoxicity (Fig. 3d) [192].

In addition, tumor-derived TGF-β suppresses NK cell mTOR signaling, thereby reducing granule release (Fig. 3c) [193]. The oncoprotein MUC1-C epigenetically silences MICA/B expression via H3K27 and DNA methylation, reducing NK recognition and subsequent granzyme-mediated killing [194]. Chronic TGF-β exposure in theTME drives NK cell exhaustion, decreases granzyme B expression, and markedly impairs cytotoxicity [195].

In summary, tumor cells employ diverse mechanisms-including disruption of apoptotic signaling, neutralization of NK effector molecules, metabolic adaptation, and cytokine-driven immunosuppression-to resist granule-mediated killing. These resistance pathways represent critical barriers to NK-based immunotherapy.

### 4.2 Aberrant FAS/FASL expression by tumor cells

FAS (Apo-1/CD95) and its ligand FASL (CD95L) are type I and II transmembrane proteins of the tumor necrosis factor (TNF) superfamily. Engagement of FAS by FASL transmits a “death signal” that activates caspase cascades and induces apoptosis in target cells. The FAS/FASL axis therefore represents a key pathway by which NK cells eliminate malignant cells [196]. However, accumulating evidence indicates that advanced tumors frequently downregulate FAS while upregulating FASL, creating an immune-privileged niche that enables evasion from NK- and cytotoxic T cell-mediated killing [197]. Thus, the FAS/FASL axis functions as a double-edged sword, with context-dependent roles in tumor cell apoptosis and immune escape.

#### 4.2.1 Downregulation of FAS expression

Loss of FAS expression allows tumor cells to evade NK-mediated apoptosis. In colorectal cancer, normal epithelial cells display FAS on the basolateral membrane, but FAS expression is reduced in a subset of adenomas and nearly absent in ∼40% of cancers. In liver metastases, expression patterns mirror those of the primary tumor [198]. Similarly, melanoma cells suppress FAS to avoid apoptosis; restoring FAS expression via gene transfection or histone deacetylase inhibition (e.g., trichostatin A) enhances NK-mediated killing [199]. In contrast, some studies suggest that FAS overexpression in colon cancer promotes tumor growth and metastasis, while its downregulation reduces liver metastasis rates [200]. In extranodal NK/T-cell lymphoma (ENKTL), ∼50% of patients harbor FAS deletions or mutations, disrupting death-inducing signaling complex (DISC) formation and conferring resistance to FAS-mediated apoptosis [201]. Mechanistically, the transcriptional repressor YY1 negatively regulates FAS expression, whereas nitric oxide donors or RKIP overexpression can restore FAS by inhibiting YY1, sensitizing tumor cells to FASL-induced apoptosis. These findings highlight FAS downregulation as a key mechanism of tumor immune escape [202].

#### 4.2.2 Upregulation of FASL expression

In contrast, many malignancies-including melanoma, colorectal cancer, sarcoma, lymphoma, hepatocellular carcinoma, and fibrosarcoma-exhibit high levels of FASL [[203], [204], [205], [206], [207]]. Tumor-expressed FASL can bind FAS on NK cells and CD8^+^ T cells, directly inducing apoptosis of effector lymphocytes and weakening antitumor immunity. This phenomenon, known as the “FAS counterattack,” is a well-established immune evasion mechanism. Beyond inducing immune cell apoptosis, FASL activates oncogenic signaling pathways such as NF-κB, MAPK, and PI3K, thereby promoting tumor proliferation, migration, and invasion [208,209].

Recent findings extend this mechanism to systemic tumor progression. High levels of FASL were detected on circulating tumor cells (CTCs) in patients with metastatic breast cancer, where FASL expression correlated with stem cell-like phenotypes and immune checkpoint expression. These observations suggest that FASL contributes not only to local immune escape but also to systemic dissemination and metastatic progression [210].

## 5 Research progress in NK cell immunotherapy

In light of the critical role of natural killer cells in the anti-tumor process, NK cells-based immunotherapy has emerged as a prominent research focus over the past decades. The main focus is on developing treatment methods that can restore the anti-tumor activity of NK cells, which include adoptive NK cells transfer therapy, immune checkpoint blockers, and chimeric antigen receptor CAR-NK cells therapy.

### 5.1 Adoptive NK cell transfer therapy

In recent years, adoptive NK cell transfer therapy has emerged as a promising immunotherapeutic approach, demonstrating considerable clinical potential. This strategy involves the infusion of NK cells derived from peripheral blood, hematopoietic stem cells, or induced pluripotent stem cells (iPSCs) into patients, with the goal of augmenting both the number and functional activity of NK cells in vivo. Adoptive NK cell transfer can be broadly categorized into autologous and allogeneic therapies. Notably, NK cells expanded ex vivo through short- or long-term activation protocols exhibit potent antitumor efficacy, with low rejection rates and minimal adverse effects [211,212].

**Fig. 4:**
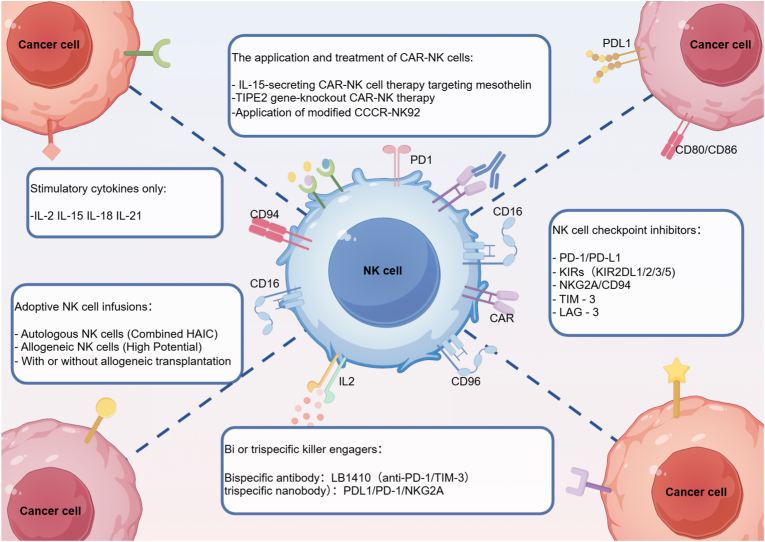
**Strategies for enhancing NK cell anti-tumor activity.** The anti-tumor efficacy of NK cells can be improved through multiple approaches: Adoptive NK cell infusions, including autologous NK cells (combined with HAIC), allogeneic NK cells (with high potential), and infusions with or without allogeneic transplantation; NK cell checkpoint inhibitors targeting molecules such as PD-1/PD-L1, KIRs (KIR2DL1/2/3/5), NKG2A/CD94, TIM-3, and LAG-3; Advanced applications of CAR-NK cells, including IL-15-secreting CAR-NK cell therapy targeting mesothelin, TIPE2 gene-knockout CAR-NK therapy, and application of modified CCCR-NK92; Administration of stimulatory cytokines like IL-2, IL-15, IL-18, and IL-21; Bi or trispecific killer engagers, including Bispecific antibody: LB1410 (anti-PD-1/TIM-3) and trispecific nanobody: PDL1/PD-1/NKG2A.

Clinical trials have advanced NK cell transfer therapies stimulated by cytokines including IL-2, IL-15, IL-18, and IL-21, underscoring their translational relevance [213](Fig. 4). In one study, NK cells were isolated from donor peripheral blood mononuclear cells of 13 patients with stage III-IV advanced lung cancer, expanded ex vivo, and infused back into the patients. Remarkably, 84.6% of participants achieved disease remission following NK cell therapy [214].

Since 2022, clinical progress in adoptive NK cell therapy has accelerated. A phase I study conducted in South Korea (June 2022) reported that in patients with locally advanced HCC refractory to standard treatment, the combination of local high-dose autologous NK cell infusion and hepatic arterial infusion chemotherapy (HAIC) achieved an objective response rate (ORR) of 63.6%, a disease control rate (DCR) of 81.8%, and a median overall survival (mOS) of 41.6 months, without serious adverse events. These findings support both the safety and efficacy of this combined approach [215](Fig. 4). More recently, on May 26, 2024, a case report described a patient with gastric adenocarcinoma and pancreatic tail metastasis who achieved disease control, with shrinkage and even disappearance of metastatic lesions, after treatment with combined NK cell therapy and chemotherapy [216].

In parallel, growing evidence supports the efficacy of allogeneic NK cell adoptive transfer, which has demonstrated therapeutic benefit across multiple cancer types. Collectively, these studies reinforce the translational promise of NK cell adoptive transfer in oncology. Nonetheless, key challenges remain, particularly in the context of solid tumors. Limited NK cell infiltration into the TME, coupled with the profoundly immunosuppressive milieu created by tumor cells, continues to significantly attenuate therapeutic efficacy.

### 5.2 Immune checkpoint molecule blockade therapy

As noted above, NK cells within the TME often exhibit high expression of inhibitory receptors. This observation has guided the development of inhibitors designed to block immune checkpoint molecules such as PD-1, TIM-3, TIGIT, and NKG2A on NK cells [217](Fig. 4). Targeted blockade of these receptors or their ligands using monoclonal antibodies (mAbs) can restore NK cell antitumor function. For example, blockade of the PD-1/PD-L1 axis enhances NK cell cytotoxicity and cytokine secretion, thereby suppressing tumor growth [218]. Several PD-1 mAbs (e.g., nivolumab, pembrolizumab) and PD-L1 mAbs (e.g., durvalumab, atezolizumab, avelumab) have been approved by the FDA for treating a range of malignancies, including melanoma, hepatocellular carcinoma, non–small cell lung cancer, and hematologic cancers [219]. While combination strategies involving two checkpoint inhibitors may yield improved clinical efficacy, they also increase the risk of immune-related adverse events.

Recent advances continue to expand the therapeutic landscape. On March 24, 2025, a study published in *Nature Communications* reported the development of a protease-cleavable PD-L1 antibody liposome (eLipo anti-PD-L1). This construct is cleaved by endogenous enzymes in the colon cancer microenvironment, enabling simultaneous immune checkpoint blockade and localized chemotherapy, and showed significant therapeutic efficacy in orthotopic colon cancer models [220].

Other inhibitors targeting NK cell checkpoints are also under active development. IPH2101, a mAb against KIR2DL1/2/3, blocks HLA-C–mediated inhibitory signaling. Preclinical studies demonstrated that IPH2101 enhances NK cell activity and survival in AML by disrupting HLA-KIR interactions [221,222]. In clinical trials, melanoma patients treated with IPH2101 showed increased NK functional capacity through reduced surface expression of KIR2D [223]. Lirilumab (IPH2102) and monalizumab (IPH2201) target NKG2A to antagonize HLA-E–mediated inhibition. Phase I/II trials are currently evaluating the efficacy of IPH2201 across tumor types. Combination strategies have shown promise: IPH2101 plus rituximab enhanced NK-mediated killing in lymphoma models [224], while IPH2201 combined with cetuximab reduced recurrence rates in head and neck squamous cell carcinoma, achieving a remission rate of 31% without significant toxicity [225]. More recently, KIR2DL5, a newly identified NK inhibitory receptor that binds PVR on tumor cells, has emerged as a therapeutic target. Anti-KIR2DL5 mAbs significantly enhanced NK cytotoxicity and survival in humanized tumor models of colorectal, ovarian, and lung cancer [226].

TIM-3 is another checkpoint of interest. Sabatolimab (MBG453), a humanized IgG4κ antibody targeting TIM-3, is being evaluated in myelodysplastic syndromes (MDS) and AML. It exhibits dual activity by targeting myeloid leukemia cells and enhancing immune cell function. Combined with PD-1 blockade, sabatolimab also shows activity in solid tumors such as melanoma and NSCLC [227]. A 2023 Ib-phase trial by Novartis reported that sabatolimab plus azacitidine/decitabine was well tolerated in high-risk MDS, achieving an overall response rate ORR of 58% (including 25% complete remission), with improved outcomes in patients with high Galectin-9 expression [228]. Another TIM-3 antibody, cobolimab (TSR-022), developed by GSK, has shown preliminary efficacy in NSCLC, liver cancer, melanoma, and leiomyosarcoma, where partial responses and disease stabilization were observed [229,230].

The LAG-3 pathway is also advancing rapidly. Relatlimab, an anti-LAG-3 antibody, was evaluated in the RELATIVITY-047 phase III trial in combination with nivolumab for advanced melanoma. The initial 2021 results demonstrated improved progression-free survival (PFS) compared with nivolumab alone [231]. Updated 33.8-month follow-up data (2025) showed that relatlimab plus nivolumab significantly prolonged both PFS (10.2 vs. 4.6 months; HR = 0.79) and overall survival (OS) (51.0 vs. 34.1 months; HR = 0.80), with grade 3–4 treatment-related adverse events reported in 22% of patients [232].

Looking forward, the design of bispecific and trispecific antibodies targeting multiple tumor antigens is underway, aiming to maximize NK cell activation while reducing immune escape [233].In the realm of bispecific drugs, LB1410, an anti-PD-1/TIM-3 bispecific antibody, has achieved complete tumor remission in the treatment of cervical cancer patients with resistance to fifth-line therapies. Its ORR as a monotherapy for immunotherapy-resistant cervical cancer reaches 38.5%. This agent can not only reverse T-cell exhaustion but also enhance NK cell activity to amplify antitumor immune responses. Currently, phase II/III clinical trials for LB1410 are underway [234].

In the field of trispecific drugs, a PDL1/PD-1/NKG2A trispecific nanobody developed by a research team from South China University of Technology has demonstrated prolonged tumor retention time in 4T1 breast cancer and B16F10 melanoma models. Specifically, the intratumoral drug retention time was 2.3-fold longer than that observed in the PD-1 monoclonal antibody-treated group. In the 4T1 breast cancer model, the tumor growth inhibition rate reached 72.6%. In the B16F10 melanoma model, the median survival time of mice was extended from 28.5 days in the control group to 56.8 days, with a complete tumor regression rate of 35% [235]. Mechanistically, this trispecific nanobody exerts potent antitumor effects by increasing CD8^+^ T-cell infiltration and activating NK cells, without inducing abnormalities in liver or kidney function [236](Fig. 4).

### 5.3 CAR-NK therapy

The clinical success of CAR-T technology has paved the way for gene-edited immune cell therapies, and building on this foundation, CAR-NK cells—generated by genetically engineering NK cells—have emerged as a promising research focus. *In vitro*, NK cells are typically expanded and functionally enhanced through stimulation with cytokines or genetic modification (e.g., the introduction of chimeric antigen receptors [CARs]). The expanded NK cells are then reinfused into patients for tumor immunotherapy [237]. In principle, CAR-modified NK cells can efficiently recognize tumor cells and eliminate them through multiple mechanisms, including the release of cytotoxic mediators and the induction of apoptosis [238,239].Compared with CAR-T therapy, CAR-NK therapy carries a lower risk of severe cytokine release syndrome (CRS), suggesting a superior safety profile for clinical use [240]. However, NK cells exhibit a shorter in vivo lifespan than T cells, resulting in reduced persistence of CAR-NK cells relative to CAR-T cells [241]. Although this may limit efficacy, it also minimizes the risks associated with prolonged survival of gene-edited immune cells, such as off-target toxicity. Consequently, CAR-NK therapy is increasingly viewed as a novel and safer approach to cancer immunotherapy.

A 2025 research report indicated that a mesothelin (MSLN)-targeted CAR-NK cell therapy engineered to secrete IL-15 prolonged the in vivo survival time of the cells to 35 days via an autocrine IL-15 signaling pathway. Fluorescent labeling tracking by the research team showed that the fluorescent signal of conventional CAR-NK cells almost disappeared within 14 days in immunodeficient tumor-bearing mice, whereas the engineered CAR-NK cells still exhibited strong fluorescent signals detectable at day 35. Analyses of tumor-infiltrating sites revealed that the CAR-positive rate of CAR-IL-15-iNK cells remained consistently above 95%. ELISA detection demonstrated that the secretion level of IL-15 was stably maintained at 80-120 pg/mL. This CAR-NK cell therapy significantly improved the tumor clearance rate in ovarian cancer models (triple that of conventional CAR-NK cells), without triggering CRS or graft-versus-host disease (GVHD), thus showing favorable safety profiles [242].

In the same year, Zhigang Tian's research team published in the *Journal for ImmunoTherapy of Cancer* a study linking TIPE2 expression to NK cell exhaustion. Using single-cell transcriptomics and gene-reporter mice, they showed that TIPE2 expression correlates with functional exhaustion of NK cells in both human and murine tumors. By knocking out TIPE2 in mouse NK cells and in human NK cells (derived from peripheral blood or iPSCs via CRISPR/Cas9), they demonstrated enhanced antitumor activity, increased tumor infiltration, and improved effector functions. Clinically, high TIPE2 expression in NK subsets was associated with poor patient survival. These findings highlight TIPE2 as a potential target for engineered NK cells and expand opportunities in “synthetic immunology” [243].

Tian's group has also reported encouraging clinical outcomes. In OC patients with a predicted survival of only 2-6 months, NK010 therapy achieved remarkable results in one case, extending survival beyond 40 months. Imaging showed complete disappearance of a 1.9 cm rectal lesion and reduction of a 1.3 cm paracolic lesion to 0.4 cm. In 2022, another study using CAR-NK therapy (CCCR-NK92) achieved a 27% reduction in left lung lesions in a patient with metastatic lung cancer [244]. That same year, a South Korean team combined allogeneic NK cells with PD-1 blockade, achieving partial response in a patient with liver metastases-tumor burden decreased by 70% in liver lesions and by 82.3% in lymph nodes, with PFS maintained for 18 months [245]. Together, these cases provide compelling evidence of the clinical potential of CAR-NK therapy.

In conclusion, CAR-NK therapy represents a promising and rapidly advancing field of immunotherapy. Its inherent safety advantages and encouraging early clinical outcomes underscore its potential. However, challenges such as limited in vivo persistence and the immunosuppressive TME remain critical barriers. Addressing these limitations will be essential for translating CAR-NK therapy into broader clinical practice.

## 6 Summary and outlook

Our understanding of NK cell–mediated antitumor activity has progressed substantially-from the earliest clinical evidence of NK cell cytotoxicity to recent advances in CAR-NK cell therapies [208,209]. Over this period, numerous preclinical and clinical investigations have explored strategies to harness NK cells for cancer treatment, ranging from in vivo cytokine stimulation to ex vivo expansion and genetic engineering.

Despite these advances, the clinical impact of NK cell-based immunotherapies remains limited. Relatively few patients have derived durable benefit, largely because NK cells, while exhibiting robust biological activity, have yet to demonstrate consistent long-term efficacy in clinical practice. Many approaches-including pharmacologic immunomodulation, adoptive transfer, and engineered NK platforms-have shown promise in preclinical models but require further validation through well-designed clinical trials.

To achieve meaningful progress, deeper insights into NK cell biology are essential. This includes elucidating the balance between activating and inhibitory receptor signaling and clarifying the complex interactions among tumor cells, NK cells, the TME, and host immune regulatory networks. A more comprehensive understanding of these mechanisms will inform the design of next-generation NK cell–based therapies and optimize their clinical translation.

## 7 Conclusion

Tumor evasion of NK cell–mediated immunity is a multifactorial process involving immunosuppressive microenvironments, upregulation of inhibitory checkpoint molecules, altered receptor–ligand dynamics, and resistance to apoptosis. Recent advances in NK cell-based immunotherapies-including adoptive transfer, immune checkpoint blockade, and CAR-NK platforms-underscore the therapeutic potential of NK cells in oncology. However, significant challenges remain, particularly with respect to enhancing NK cell persistence, trafficking, and functional activation within the TME. Addressing these barriers through mechanistic insights and innovative therapeutic strategies will be critical to translating NK cell-based therapies into durable clinical benefit.

## Author contribution information

Rulin Zheng, Zhuqing Wang, Baodi Zhang and Yiwei Li contributed equally to this work. Rulin Zheng, Zhuqing Wang, Baodi Zhang and Yiwei Li performed the literature review and wrote the manuscript. Shan Gao and Yuhan Guo supplemented and improved the content. Liwei Shao, Xiaodong Mu and Deping Meng supervised the project and revised the manuscript. All authors read and approved the final manuscript.

## Ethics approval

Not applicable.

## Declaration of generative AI in scientific writing

Not applicable.

## Funding information

This work was supported by grants from the 10.13039/501100007129Shandong Provincial Natural Science Foundation (ZR2023QH356).

## Declaration of interest statement

All authors who have actively participated in this study agree with the submission of the manuscript to Cancer Treatment Reviews. The work has not been published elsewhere, either completely, in part, or in another form, and that the manuscript has not been submitted to another journal and will not be published elsewhere within one year after publication in this journal. There is no any potential conflict of interest. The authors do not have commercial or other associations that might pose a conflict of interest.

## Data Availability

Not applicable.
